# Serum microRNA qPCR profiling and validation indicate upregulation of circulating miR-145-5p and miR-26a-5p in migraineurs

**DOI:** 10.1186/s10194-024-01908-x

**Published:** 2024-11-18

**Authors:** Joanna Kordacka, Renata Gruszka, Magdalena Zakrzewska

**Affiliations:** 1 Department of Molecular Pathology and Neuropathology, Medical University of Lodz, Lodz, Poland; 2 Department of Molecular Biotechnology and Genetics, Faculty of Biology and Environmental Protection, University of Lodz, Lodz, Poland

**Keywords:** Biomarkers, Epigenetics, Migraine, microRNA, miRNA

## Abstract

**Background:**

In recent years, miRNAs found in biological fluids have gained interest as biomarkers of numerous conditions, including migraine. This study aimed to identify differences in the levels of circulating miRNAs in the serum of migraineurs as compared to healthy controls, as well as between patients with different types of migraine and during the ictal and nonictal phases of the condition.

**Methods:**

The screening phase of the study included serum from 13 migraine patients and 13 sex and age matched controls. A panel of 179 miRNAs was analysed using locked nucleic acid SYBR based qPCR. Based on statistical analysis (U Mann-Whitney test) and data from existing literature, nine miRNAs were selected for validation by TaqMan qPCR in an independent cohort of 26 migraineurs and eleven healthy controls. For comparison between the study and control group, U Mann-Whitney test was performed. The differences between patients with chronic and episodic migraine, migraine with and without aura and in ictal and nonictal phases were analysed with Kruskal-Wallis test. The results were corrected for multiple comparisons using Benjamini-Hochberg method. In all analysis p value ≤ 0,05 was considered as significant.

**Results:**

Two miRNAs, miR-145-5p and miR-26a-5p were significantly upregulated in serum of migraineurs compared to healthy controls. MiRNA-19a-3p was downregulated in patients currently experiencing migraine headache compared to those in the interictal period. No differences were found between patients with different migraine types.

**Conclusion:**

The results of our study add to the growing body of evidence for dysregulation of the circulating miRNA profile by migraine. They are further supported by previous reports on differential expression of miR-145-5p, miR-26a-5p and miR-19a-3p in migraineurs. However, more research on larger populations is needed to validate these findings, as well as elucidate the role of circulating miRNAs in the condition. Moreover, to wholly explore the biomarker potential of miRNAs, migraine patients should not only be compared to healthy controls but also to populations with different headache disorders.

**Graphical Abstract:**

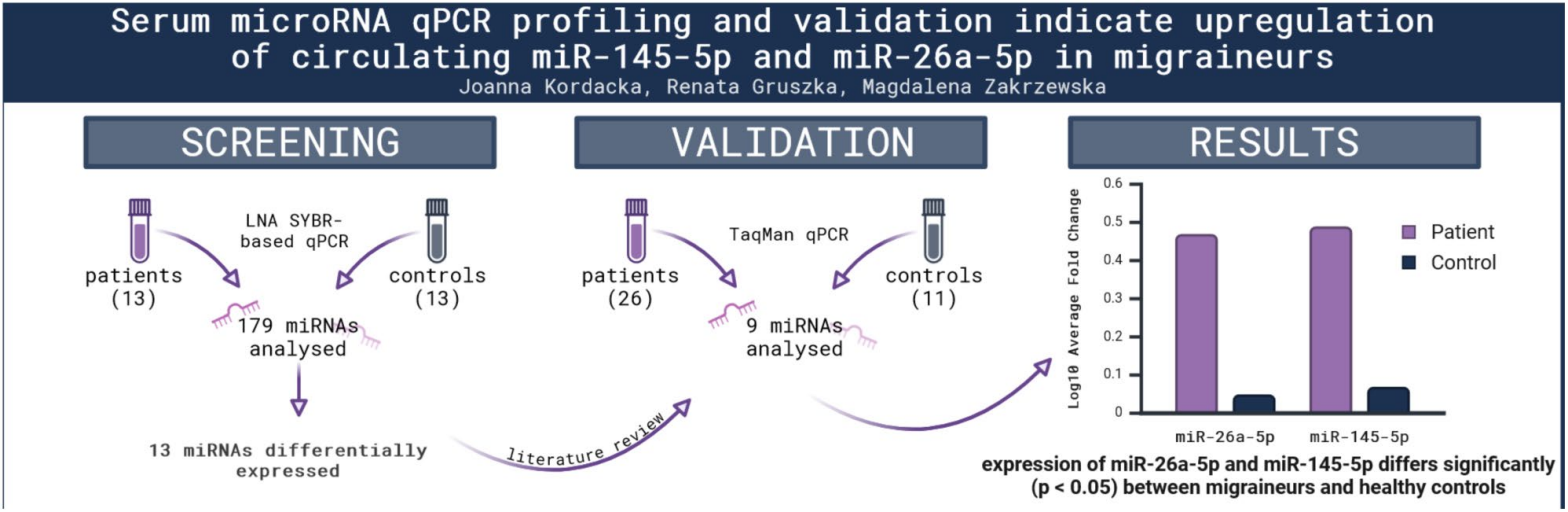

**Supplementary Information:**

The online version contains supplementary material available at 10.1186/s10194-024-01908-x.

## Introduction

Migraine is, after tension-type headache, the second most common type of primary headache, affecting about 15% of the population worldwide [1], causing not only physical pain but also profoundly influencing the personal and professional lives of those affected [2].

Still, there are no established, objectively measurable biomarkers of the disorder, and the diagnosis is made solely based on clinical criteria. Although the definitions in ICHD-3 are very precise and the classification contains guidelines that dispel doubts in unclear situations, there is still room for miscommunication between the doctor and the patient or doubt in the face of more complex, ambiguous symptoms. This may lead to costly additional tests, diagnostic delays or misdiagnoses [3].

In recent years, circulating miRNAs have been proposed as potential biomarkers of numerous conditions, including migraine [4, 5].

MicroRNAs (miRNAs) are a type of small non-coding RNAs that play a vital role in post-transcriptional regulation of gene expression. They are not only found in intracellular space but can also be released into body fluids, such as blood, saliva, urine, milk or semen [6]. Notably, the type and quantity of miRNAs released do not necessarily reflect the miRNA profile of the cell of origin, suggesting that the particles found in the bloodstream are not merely waste products but are selectively exported outside the cell [7]. Consequently, a better understanding of the miRNA landscape in migraine may allow us to gain greater insight into the pathophysiology of the condition. Therefore, circulating miRNAs have been studied not only as markers facilitating the diagnosis of migraine but also as indicators of the headache phase itself or predictors of treatment response [4, 8–11].

In this study, we investigated the expression levels of a wide panel of serum miRNAs in migraineurs and compared them to healthy controls. We also looked for differences between patients in the ictal and interictal phases, those experiencing and not experiencing aura, and those with episodic and chronic migraine.

## Materials and methods

### Study population

Two independent patient cohorts took part in the screening and validation stages of the study. The characteristics of each group of migraineurs are summarised in Table 1. All subjects included in the study were adults (18 years old or older). All patients signed an informed consent prior to blood sampling. The diagnoses were made according to the third revision of the International Classification of Headache Disorders (ICHD-3) in either the outpatient neurology clinic, the Clinical Department of Neurology or the Emergency Room of the Norbert Barlicki Memorial Teaching Hospital No. 1 of the Medical University of Lodz.

Sample collection during the ictal phase was defined as any time the patient reported experiencing migraine headache, regardless of its duration. The nonictal samples were obtained from patients declaring complete freedom of migraine symptoms. None of the samples was obtained during the aura. Data about comorbidities and medication can be found in Supplementary Material 1.

The exclusion criteria were:

- Migraine prophylaxis at the time of blood sampling.
- Infectious processes at the time of blood sampling.
- Malignancy at present and in past medical history.
- Other neurological conditions, excluding back pain and asymptomatic arachnoid cysts or granulations as accidental finds on brain imaging.
- Pregnancy.

In both stages of the analysis, we used biobanked serum from Biobank HARC of the Medical University of Lodz. In the profiling stage, 13 controls were sex and age matched to the migraine patients. In the validation stage of the study, in order to obtain the closest possible age match we utilised two of the controls used at the previous stage, as well as nine independent control samples. All control subjects signed an informed consent prior to the deposition of their biological material.

**Table 1:** Characteristics of the study cohorts

	Screening cohort	Validation cohort
Number	13	26
Female: male	11:2	23:3
Age (years)	24–63, median: 43, SD: 11.64	19–66, median: 40, SD: 12.31
Diagnoses	EM: 8 EMA: 4 CM: 1	EM: 11 EMA: 10 CM: 3 CMA: 2
Nonictal: ictal phase	9:4	21:5

CM – chronic migraine without aura, CMA – chronic migraine with aura, EM – episodic migraine without aura, EMA – episodic migraine with aura, SD – standard deviation

### Serum sampling

The blood samples were extracted from one of the veins of the arm and collected into 7.5-mL S-Monovette^®^ Serum CAT with a clotting activator (Sarstedt AG & Co., Nümbrecht, Germany). The test tubes were inverted 3–6 times and then left for coagulation for 30 min and consequently centrifuged at 2000 x g for 10 min at room temperature. Depending on its volume the supernatant was transferred into 3–4 smaller tubes in 200–600 µl aliquots and immediately frozen at -80 °C until further processing.

### miRNA isolation and cDNA synthesis

Total RNA, including miRNA, was isolated from 200 µL of serum using the miRNeasy Serum/Plasma Advanced Kit (cat. no. 217204, Qiagen, Germany) according to the manufacturer’s protocol. All RNA samples were stored at -80 °C until further analysis.

During the profiling step, each sample was transcribed into cDNA using the miRCURY LNA RT Kit (cat. no. 339340, Qiagen, Germany). The UniSp6 RNA spike-in was used as a reverse transcription positive control.

During the validation step, TaqMan™ MicroRNA Reverse Transcription Kit (cat. no. 4366596, Applied Biosystems) was used.

### Screening

For each profiling sample the miRCURY LNA miRNA QC PCR Panel (cat. no. 339331, Qiagen, Germany) was used for quality control. Only optimal samples were included in the Quantitative PCR (qPCR) profiling. miRCURY LNA miRNA PCR Panel for serum and plasma analysis (cat. no. 339325, Qiagen, Germany) was used in 96-well PCR format with a total of 179 LNA miRNA primers (Supplementary Material 2). UniSp3 was used as an inter-plate calibrator. The target cDNA sequences were amplified by qPCR in a CFX Connect Real-Time PCR System (Bio-Rad, California, USA).

### Validation

After analysing the profiling results and conducting a literature review, we selected miRNAs (Table 2) for investigation using TaqMan probes during validation (cat. no. 4427975, Life Technologies, Carlsbad, CA, USA). For each sample, a fixed volume of 3µL of RNA was used in reverse transcription with TaqMan™ MicroRNA Reverse Transcription Kit (cat. No. 4366596, Thermo Fisher Scientific Baltics UAB, Lithuania). Each qPCR was performed in duplicate on every individual migraine/control sample using TaqMan™ Fast Advanced Master Mix for qPCR (cat. no. 4444557, Thermo Fisher Scientific Baltics UAB, Lithuania).

**Table 2:** List of miRNAs analysed by qPCR during validation experiment

	No	miRNA name	Assay ID	miRNA target sequence
Validated miRNAs	1	hsa-miR-145-5p	**002278**	GUCCAGUUUUCCCAGGAAUCCCU
	2	hsa-miR-26a-5p	**000405**	UUCAAGUAAUCCAGGAUAGGCU
	3	hsa-miR-27b-3p	**000409**	UUCACAGUGGCUAAGUUCUGC
	4	hsa-miR-126-3p	**002228**	UCGUACCGUGAGUAAUAAUGCG
	5	hsa-miR-34a-5p	**000426**	UGGCAGUGUCUUAGCUGGUUGU
	6	hsa-miR-19a-3p	**000395**	UGUGCAAAUCUAUGCAAAACUGA
	7	hsa-miR-424-5p	**000604**	CAGCAGCAAUUCAUGUUUUGAA
	8	hsa-miR-19b-3p	**000396**	UGUGCAAAUCCAUGCAAAACUGA
	9	hsa-miR-125a-5p	**002198**	UCCCUGAGACCCUUUAACCUGUGA
Control miRNAs	10	hsa-miR-18a-5p	**002422**	UAAGGUGCAUCUAGUGCAGAUAG
	11	hsa-miR-652-3p	**002352**	AAUGGCGCCACUAGGGUUGUG
	12	hsa-miR-335-5p	**000546**	UCAAGAGCAAUAACGAAAAAUGU
	13	hsa-miR-93-3p	**002139**	ACUGCUGAGCUAGCACUUCCCG
	14	hsa-miR-451a	**001141**	AAACCGUUACCAUUACUGAGUU
	15	hsa-miR-23a-3p	**000399**	AUCACAUUGCCAGGGAUUUCC

### Analysis and statistical analysis

All statistical analyses were performed in Excel (Microsoft, Redmond, WA, USA) and STATISTICA v. 12 (StatSoft Polska Sp. z o.o., Krakow, Poland).

#### Screening stage

Quality control and calibration of the data from the qPCR were done using GeneGlobe Data Analysis Software (Qiagen, Germany). Three miRNAs were chosen for normalisation based on their consequent expression across samples, and the stability factor was determined using NormFinder (hsa-miR-93, hsa-miR-18b-5p, hsa-miR-18a-5p). The ΔΔCq method was used to determine fold change, as described by Livak and Schmittgen [12] and Riedel et al. [13]. The conducted equations can be seen below:

$$\:\varDelta\:Cq={Cq}_{Target}-{Cq}_{Avg\left(Reference\:miRNAs\:Cq\right)}$$

$$\:\varDelta\:\varDelta\:Cq=\varDelta\:{Cq}_{Target}-\varDelta\:{Cq}_{Avg(Control\:Group\:\varDelta\:Cq)}$$

$$\:Fold\:change={2}^{-\varDelta\:\varDelta\:Cq}$$

Where Cq_Avg(Reference miRNAs Cq)_ is the arithmetic mean of Cq values of hsa-miR-93, hsa-miR-18b-5p and hsa-miR-18a-5p and ΔCq_Avg(Control Group ΔCq)_ is the arithmetic mean of ΔCq values of the target miRNA in the control group.

The Shapiro-Wilk normality test showed that not all data followed normal distribution. Both log transformation and Box-Cox transformation resulted neither in normal distribution nor homogeneity of variance of all data. Therefore, more powerful, parametric statistical tests could not be performed. Consequently, the differences between the groups were analysed with U Mann-Whitney test. P value ≤ 0,5 was considered as significant.

#### Validation stage

We determined the fold change using the same method described above. As we used a different qPCR technology to validate the data, we again performed a NormFinder analysis and chose miR-93-3p, miR-18a-5p and miR-23a for normalisation, based on their stability factor.

The data from the validation cohort was normally distributed. However, in our opinion, at this stage of analysis, the power to detect minuscule differences was less significant than the precision of the results and avoidance of false positives, which were likely to appear in a small experimental sample like ours. Therefore, we decided to utilise the U Mann-Whitney test again for the comparison between migraine and control groups as well as between chronic and episodic migraine, migraine with and without aura and ictal and nonictal phases. For the comparison between the subgroups: episodic migraine without aura, episodic migraine with aura, chronic migraine without aura, chronic migraine without aura Kruskal-Wallis test was used. In all analysis p value ≤ 0,05 was considered as significant.

### In silico target prediction and search for experimentally validated targets

We used miRWalk [14] for in silico target prediction of the miRNAs differentially expressed in our experiment. To ensure high accuracy, we filtered the results only to include targets predicted simultaneously by at least one more prediction system, either TargetScan [15] or miRDB [16]. For searching for experimentally validated miRNA-target interactions, miRTarBase [17] was used. The resulting dataset was screened for targets that had been shown to be expressed in the trigeminal ganglion [18, 19], as well as migraine susceptibility loci identified in genome-wide association studies (GWAS) [20, 21].

## Results

**Fig. 1:**
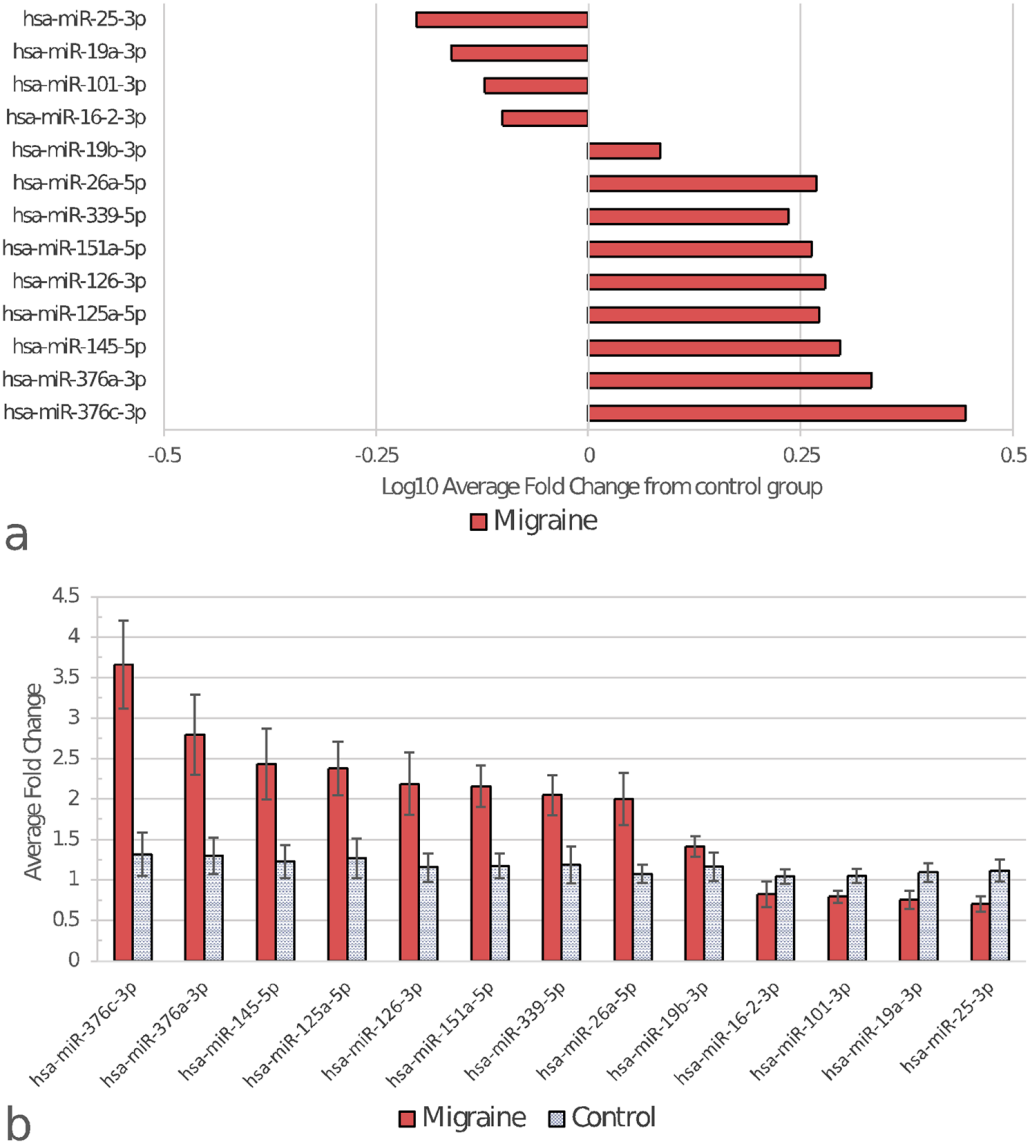
miRNAs significantly dysregulated in migraineurs as indicated by the screening analysis. For fold change determination method see Materials and Methods section. (**a**) Log10 representation of average fold change of the migraine samples, normalized to control. Zero on the X scale indicates control average. (**b**) Raw average fold change values of migraine and control samples, with error bars indicating the standard error of the mean

The initial screening analysis identified 13 miRNAs as potentially differentiating between migraineurs and healthy controls (Fig. 1). Out of these, six were selected for further analysis based on the magnitude of fold change and literature review, with priority given to miRNAs that had been found to differ in expression in previous studies, whether at the screening or validation stage. On this basis, we also decided to include miR-424-5p and miR-27b-5p, which both showed a trend towards differential expression that did, however not reach statistical significance (*p* = 0.064 and 0.081, respectively). Finally, although miR-34a-5p was not shown to be aberrantly expressed during initial analysis (*p* = 0,8403), we decided to include it in further experiments due to the number of reports connecting it to migraine [8, 9, 11, 22].

**Table 3:** Probability values for comparisons of differences between miRNA levels in migraine and control groups at the validation stage

miRNA	*p* value	q value
miR-26a-5p	**0**,**00004**	**0**,**00028**
miR-145-5p	**0**,**00016**	**0**,**00057**
miR-126-3p	0,06046	0,14107
miR-125a-5p	0,14833	0,25958
miR-19b-3p	0,22519	0,31526
miR-19a-3p	0,80319	0,93706
miR-27b-3p	0,84598	0,84598

q value indicates p value corrected for multiple comparisons with Benjamini-Hochberg method. Statistically significant values are shown in bold

**Fig. 2:**
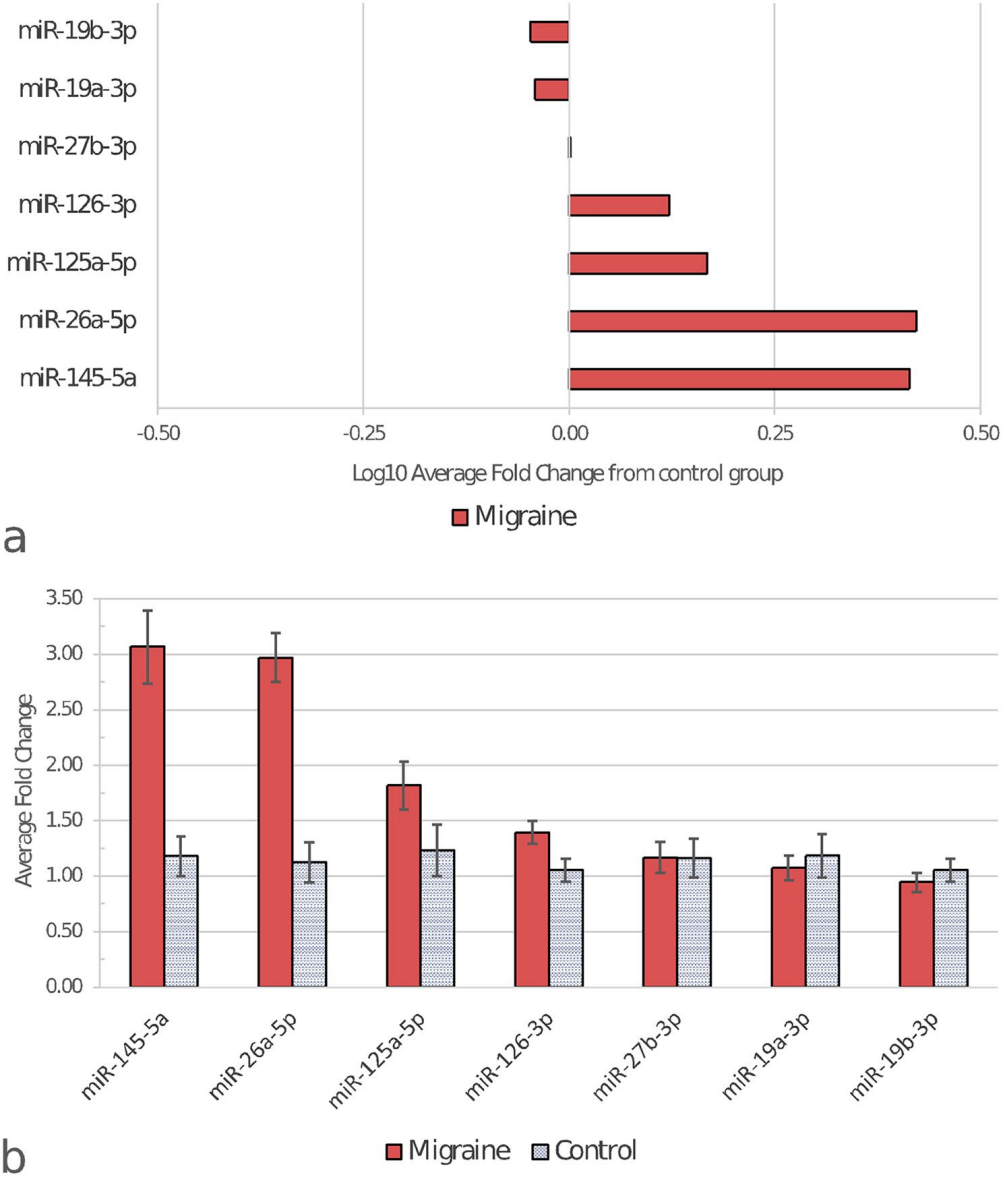
miRNAs analysed in the validation stage. For fold change determination method see Materials and Methods section. (**a**) Log10 representation of average fold change of the migraine samples, normalized to control. Zero on the X scale indicates control average. (**b**) Raw average fold change values of migraine and control samples, with error bars indicating the standard error of the mean

Among miRNAs chosen for validation, miR-145-5p and miR-26a-5p showed differential expression between migraine and control groups (Fig. 2). These differences remained significant after Benjamini-Hochberg correction for multiple comparisons (FDR 5%). A distinct tendency to differentiate these two groups was also observed for miR-126-3p but it did not reach statistical significance (Table 3). MiR-424-5p did not reach the detection threshold in any of the samples, and miR-34a-5p did not reach it in 49% of them. Therefore, these miRNAs were excluded from further analysis.

**Fig. 3:**
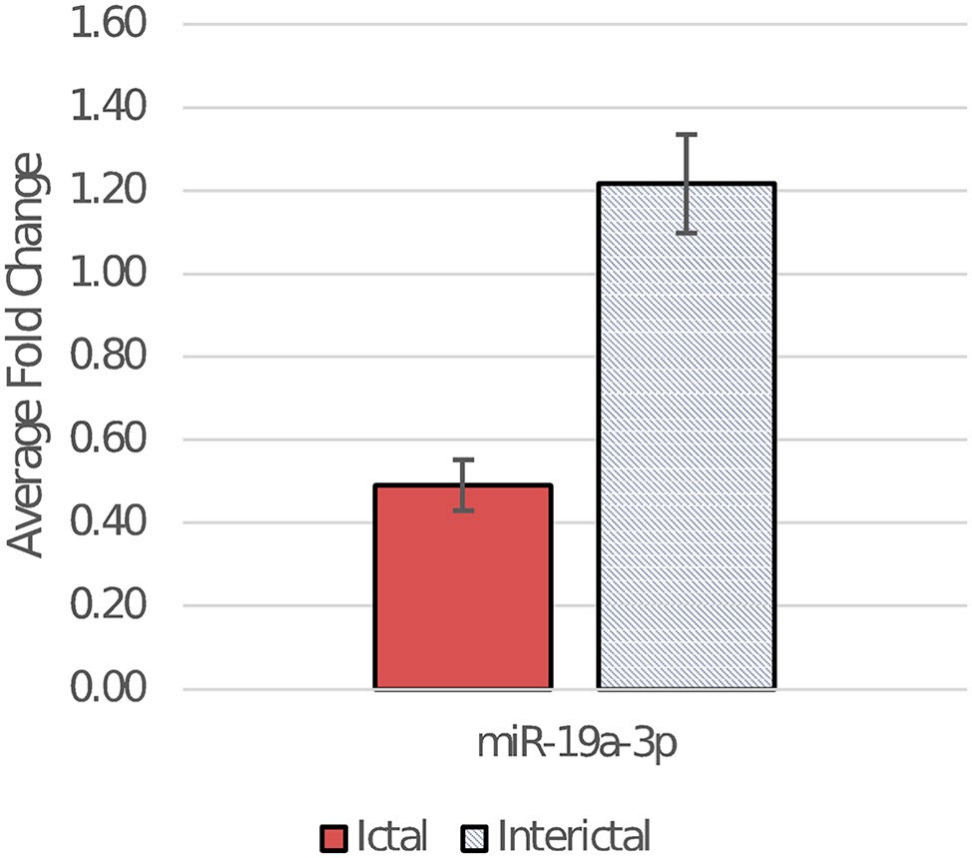
Average fold change of miR-19a-3p in ictal and interictal samples. Error bars indicate standard error of the mean. For fold change determination method see Materials and Methods section

Expression of miR-19a-3p was lower in the ictal than in the interictal group (*p* = 0.0018) (Fig. 3). No difference in miRNA expression could be shown between patients experiencing and not experiencing aura and between those with episodic and chronic migraine.

The results of in silico target prediction and search for experimentally validated targets of the miRNAs found to be differentially expressed in the study were filtered for potentially relevant interactions and are presented in Tables 4 and 5. The raw Cq values resulting from both qPCR experiments can be found in Supplementary Material 3.

**Table 4:** Predicted target genes of miRNAs differentially expressed in migraineurs

miRNA	Target	miRWalk	TargetScan	miRDB	Migraine susceptibility loci	Expressed in TG	Additional information
miR-145-5p	*ATP1A2*	×	×	×	−	×	Mutations in *ATP1A2* are causative of Familial Hemiplegic Migraine t. 2 [54]
	*MMP16*	×	×	×	−	×	SNPs reported to influence treatment response [55]
	*PLCE1*	×	×	×	×	×	SNPs reported to influence migraine treatment response [56]
	*NFIB*	×	×	−	×	−	
	*ZEB2*	×	×	−	×	−	
miR-26a-5p	*RNF213*	×	×	−	×	×	Variation in *RNF213* cause moyamoya disease-2 [57, 58]
	*TJP2*	×	−	×	×	−	
miR-19a-3p	*SCN3A*	×	×	×	−	×	Mutations cause *SCN3A*-Related Neurodevelopmental Disorder [59]
	*SCN9A*	×	×	×	−	×	Mutations in the gene cause several pain-related congenital disorders [60]

TG – Trigeminal Ganglion

**Table 5:** Validated target genes of miRNAs differentially expressed in migraineurs

miRNA	Target	Migraine susceptibility loci	Expressed in TG	Additional information
miR-145-5p, miR-26a-5p, miR-19a-3p	*TGFBR2*	×	×	TGF-β plays a role in the development of cerebral vasculature [61], some studies indicate its elevation in migraine and tension type headache [62]
	*ESR1*	−	×	
miR-145-5p, miR-19a-3p	*ABHD17C*	×	−	
miR-145-5p	*SNX24*	×	−	
miR-26a-5p	*MTDH*	−	×	Polymorphism of the regulatory region of MTDH (Rs1835740) found in both migraineurs and cluster headache patients [63]
	*TBC1D16*	×	−	Variants may predispose to moyamoya disease [64]
	*PTGS2*	−	×	Also known as COX2, encoding enzyme crucial to the inflammation and pain initiation and target of NSAIDs [65]
	*NRP1*	−	×	
miR-19a-3p	*NFIB*	×	−	
	*ZBTB4*	×	−	
	*MACF1*	×	−	
	*CAMTA1*	×	−	
	*RBM20*	×	−	

**NSAIDs** – Non-steroidal Anti-inflammatory Drugs, **TG** – Trigeminal Ganglion

.

## Discussion

The knowledge about circulating miRNAs in migraine is consequently being expanded in new studies (Table 6). When we started this project, only a few groups investigated the connection between these molecules and migraine. However, a recent systematic review by Grodzka et al., published in July 2023, highlights twenty-one works on the topic, ranging from those that explore circulating miRNAs as possible diagnostic markers to ones searching for the change in their expression after treatment to preclinical studies on animal models [4]. There is growing evidence for the involvement of epigenetic processes, including microRNA-mediated gene silencing, in various painful syndromes, many of which, such as complex regional pain syndrome, of still undetermined aetiology and unclear mechanism [5].

**Table 6:** Summary of selected aspects of works on miRNA in amorphous blood components and migraine

	Andersen et al. [11]	Cheng et al. [23]	Liu et al. [10]	Tafuri et al. [24]	Our study
Number of patients	screening: 8 (Cohort 1) validation: 20 (Cohort 1 plus 12 independent samples (Cohort 2))	30	20	15	screening: 13 validation: 26
Female: male patients	Cohort 1: 6:2 Cohort 2: no data	22:8	no specific data, randomly chosen from a cohort with F: M ratio 46:14	15:0	screening: 11:2 validation: 23:3
Material	serum	plasma	exosomes isolated from serum	exosomes isolated from plasma, PBMCs	serum
Number of miRNAs investigated	screening: 372 validation: 4	4	screening: small RNA sequencing, validation: 4	screening: 176 validation: 10	screening: 179 validation: 9
Main results	↑ ictaly: miR-34a-5p, miR-29c-5p, miR-382-5p ↑ interictally: miR-382-5p	↑ interictally: miR-155, miR-126, let-7 g	↑ interictally: miR-369-5p ↓ interictally: miR-550a-3-5p, miR-145-5p	↑ interictally: miR-27b ↓ interictally: miR-181a and miR-22	↑ independent of ictal status: miR-145-5p, miR-26a-5p ↓ ictaly: miR-19a-3p

The arrows indicate the direction of the change: ↑ - upregulation, ↓- downregulation of expression

In our work, we demonstrate two miRNAs, miR-26a-5p and miR-145-5p, to be upregulated in the serum of migraineurs compared to healthy controls and one miRNA, miR-19a-3p, to be downregulated during headache compared to the interictal period. We have not found any differences between patients with different migraine subtypes (episodic vs. chronic, with vs. without aura). However, due to the small number of patients in each category, these results should be interpreted with caution, considering that the study was not powered to detect small differences in miRNA expression.

When selecting miRNAs for further analysis in our experiment, we placed particular emphasis on those that had already been mentioned in earlier works on the topic. Some of our results confirm previous observations, while others contradict them.

During the screening phase of the study by Tafuri et al. [24], miR-26a-5p also presented differential expression between migraineurs and controls, however during the validation stage, this difference failed to prove significant (*p* = 0.0926). Interestingly, Liu et al. [10] showed miR-145-5p to be downregulated in their headache cohort, compared to healthy controls. Furthermore, a true acupuncture procedure, an intervention studied in that experiment, enhanced the expression of this miRNA species in patients who underwent it. Likewise, while both Tafuri [24] and Andersen [11] demonstrated upregulation of miRNA-424-5p at the screening stage of their work, at this phase of our experiment miRNA-424-5p appeared to be downregulated in headache patients. Due to this result, we have chosen it for further analysis using TaqMan™ probes based qPCR technique and repeatedly found it to be under the detection threshold in both patients and controls.

Similarly, while several studies point to dysregulation of miR-34a-5p levels in migraine [9, 11, 22, 25–27], in our analysis, this miRNA was not differentially expressed between study and control cohorts in the screening step. Nevertheless, we included it in the validation stage based on the weight of current proofs of its dysregulation in the condition. Still, at this step, it did not show a differential expression, failing to cross the detection threshold in many samples. The lack of difference prevailed even while comparing samples obtained during ictal- and nonictal phases, as well as patients with episodic and chronic migraine.

The reasons behind these disparities are likely multifactorial. One of them may be the differences in biological material used for analysis. While Liu and Tafuri isolated exosomes from either serum or plasma respectively, both Andersen’s and our analysis were performed without this step; however, they still differed in the blood derivatives used. Therefore, the possibility that certain miRNAs are up- or down-regulated in specific compartments while showing the opposing trend in another cannot be excluded. This idea is further supported by the comparison of miRNA signatures of exosomes and peripheral blood mononuclear cells (PBMCs) performed by Tafuri – despite being up- or down-regulated in plasma-derived exosomes, miR-27b and miR-181a did not show significant modulation by migraine in PBMCs. Another study focused solely on this problem has shown that PBMCs and whole blood differ in their miRNA profiles [28], and the results of studies using these materials cannot be directly compared to each other. Such observations are not meant to be interpreted as proof of the superiority of any biological material over another, but they merely underline the complexity of circulating miRNA biology.

We looked at circulating miRNAs primarily as diagnostic markers with the potential for practical and widespread use. In the future, such a marker could facilitate making the proper diagnosis or even the decision to implement migraine-specific treatment. Therefore, its testing should be widely available and inexpensive. For this reason, we chose serum for testing as it is an easily obtainable and economical material that does not require inaccessible preparation procedures.

The use of varied laboratory methods is also likely to play a role. In our work, different qPCR techniques, as well as laboratory kits from different manufacturers, were used at the initial screening and subsequent validation steps. This approach leads to the detection of more robust differences which do not disappear due to technical limitations of reagents or detection methods [27]. It might have led to some findings that were concordant with previous works at the screening stage, losing significance after the validation procedure. It is worth emphasizing here that independent groups of patient samples and minimally overlapping control samples were used at both steps of our analysis. Due to variability between populations as well as individuals within them, this method leads to a lower probability of confirming the results of the screening procedure while at the same time highlighting robust findings, more likely to truly arise from the studied condition [29, 30].

Another reason for this variety in the findings may lie in the differences in studied populations. Numerous works report differences in circulating miRNA levels due to both lifestyle factors and ancestry [31–35]. Although some variables like BMI, smoking status or menstrual cycle phase were controlled in individual studies, none of the works mentioned above, including ours, took note of all the factors that may influence circulating miRNA levels. However, in our opinion, such approach, while of invaluable importance during exploratory work [36], is not transferable from bench to bedside, as it would limit the usage of circulating miRNAs as diagnostic biomarkers to only specific, carefully pre-selected cases, contradicting the goal of facilitating and shortening the path to diagnosis. Possibly, until more is known about various influences on the composition of miRNA pool in the bodily fluids, circulating miRNAs may sooner find practical application as markers of treatment efficacy, where samples from a single patient are taken before and after an intervention and compared to each other, instead of being used for diagnosing purposes. Possible usability of miR-143-3p in this role was demonstrated recently by Ornello et al. [8], who showed that a greater decrease in its level in PBMCs derived from women pre- and post-treatment with erenumab correlated with better response to the drug.

Our study was not directly designed to prove any relationship between miRNAs, their targets, and migraine pathophysiology. However, to provide a basis for further exploration with the goal of elucidation of the miRNA role in the condition, we searched for predicted and validated targets of miR-145-5a, miR-26a-5p and miR-19a-3p as described in the *Materials and methods* section. In Tables 4 and 5 we present the results of screening the acquired dataset for targets shown to be expressed in the trigeminal ganglion [18, 19], as well as migraine susceptibility loci identified in genome-wide association studies (GWAS) [20, 21], together with additional data from reviewing the existing literature, if relevant. A literature search has also been performed to search for previous studies on miR-145-5a and miR-26a-5p in the context of various systems and cell types involved in the condition.

MiR-145 is abundantly expressed in tissue samples derived from both rat and human arteries and plays an important role in vascular smooth muscle cells’ differentiation and phenotype regulation [37–39]. Functional dysfunction of these cells was demonstrated in migraineurs [40, 41], and their morphological changes were shown in the biopsies of temporal and occipital arteries performed during surgical decompression of migraine trigger points [42].

Both miR-145 and miR-26 have been studied in animal and cell culture models of ischemic stroke, a condition that migraineurs may have an elevated risk for [43]. Experimental increase in the expression of miR-26a in a mouse model of ischemic stroke and neuron and microglia cell culture oxygen-glucose deprivation injury model reduced microglial inflammation and neuronal apoptosis [44]. In a similar rat model miR-145 was demonstrated to decrease apoptosis of neuronal stem cells and promote their differentiation into neuronal cells [45], an effect also demonstrated in stem cells derived from mouse foetal forebrain [46]. On the other hand, a different experiment, also using a rat model, demonstrated that overexpression of miR-145-5p caused an increased inflammatory and cytotoxic response in neurons by downregulating Nuclear receptor-related factor 1 (Nurr1) and consequentially promoting TNF-α expression in surrounding microglia [47]. Such varied and seemingly opposing effects induced by the same miRNA in one condition are, in fact, not contradictory but rather highlight one of the core characteristics of these non-coding RNAs, which is targeting multiple mRNA targets by the same miRNA. Although hypothesised to be beneficial in potential future miRNA medications for complex, multifactorial and multigenic diseases, such as neoplasms [48], this quality may also somehow limit the usefulness of single miRNAs as disease biomarkers in real-world situations.

While the strength of our study lies in the inclusion of an independent validation cohort and utilisation of two different qPCR systems, our work is also affected by limitations. The main one is the sample size, which, albeit comparable with other works on the topic, is still small, considering the prevalence of migraine in the population. The results, especially of the subgroup comparisons, must be interpreted with this precaution in mind and further validated in a larger population.

There are also other limitations to the subgroup comparisons. The sorting of the patients into chronic and episodic migraine groups was a binary division based strictly on the ICHD-3 criteria, without taking into account headache, or specifically migraine headache, frequency as a continuous variable. Thus, we cannot exclude that the groups were actually similar to each other in terms of the headache burden, which would explain the lack of significant differences between them.

The study design did not involve obtaining a serum sample twice from the same person, so the patients in the ictal phase were compared to different, headache-free patients and not to themselves. Therefore, not all confounding factors that may influence the comparison can be ruled out. Although patients with major comorbidities were excluded from the study, some of the participants had conditions that are common in the general population, as well as some regarded to coincide with migraine, such as depression [49–51] and hypothyroidism [52]. This situation better reflects the actual migraine population, nevertheless other diseases and medication intake may also affect miRNA levels and mask the impact of migraine. There are also other factors that may influence miRNAs expression in serum and that were not accounted for in our work, such as BMI, level of physical activity or smoking status [53].

Finally, though we had control over handling of the samples obtained from the migraine patients, our control samples came from a biobank and were not collected with specifically miRNA studies in mind. To limit the impact of differences in pre-laboratory handling, we adjusted our sampling protocol to match the one used by Biobank HARC of the Medical University of Lodz.

## Conclusion

With this work, we add another piece of evidence that the circulating miRNA profile is modulated by migraine. The miRNAs that were upregulated in migraineurs in our study, namely miRNA-145-5p and miRNA-26a-5p, as well as miR-19a-3p, downregulated during headache, have also been highlighted in previous works [10, 24] strengthening the plausibility of their role in the disorder. However, before circulating miRNAs can truly be utilised as diagnostic biomarkers, such studies must be repeated on significantly larger populations. Research is also missing on the comparison of migraine patients to those with other, both primary and secondary headache disorders, a differentiation possibly more important in the clinical setting. Furthermore, in order to reliably connect miRNAs to any specific pathological process, more data has to be gathered on their origins, different factors influencing their levels, as well as their multidirectional effects on multiple targets and under different conditions.

## Electronic supplementary material

Below is the link to the electronic supplementary material.

Supplementary Material 1

Supplementary Material 2

Supplementary Material 3

## Data Availability

The raw dataset on which all further analyses were performed can be found in Supplementary Material 3.

## Abbreviations

BMI: Body Mass Index
CMA: Chronic migraine with aura
CM: Chronic migraine without aura
EMA: Episodic migraine with aura
EM: Episodic migraine without aura
FDR: False Discovery Rate
GWAS: Genome-Wide Association Study
ICHD: International Classification of Headache Disorders
LNA: Locked Nucleic Acid
microRNA: micro ribonucleicacid
miRNA: micro ribonucleicacid
NSAIDs: Non-steroidal Anti-inflammatory Drugs
PCR: Polymerase Chain Reaction
qPCR: Quantitative Polymerase Chain Reaction
RNA: Ribonucleicacid
SD: Standard Deviation
TG: Trigeminal Ganglion
